# Regulatory role and molecular mechanism of METTL14 in vascular endothelial cell injury in preeclampsia

**DOI:** 10.17305/bb.2024.10963

**Published:** 2024-09-24

**Authors:** Huafang Wei, Lin Liang, Chengwen Song, Ming Tong, Xiang Xu

**Affiliations:** 1 Department of Gynaecology and Obstetrics, General Hospital of the Central Theater Command of the Chinese People’s Liberation Army, Wuhan, China; 2 Supervision Office, Changsha Health Vocational College, Changsha, China

**Keywords:** Preeclampsia, vascular endothelial cell injury, METTL14, miR-34a-5p, forkhead box protein 1, N6-methyladenosine (m6A) modification

## Abstract

Preeclampsia (PE) is a pregnancy-related disease characterized by vascular endothelial cell injury. This study aimed to investigate the role of methyltransferase-like protein 14 (METTL14) in vascular endothelial cell injury in PE. A PE cell model was established by treating human umbilical vein endothelial cells (HUVECs) with tumor necrosis factor-alpha (TNF-**α**) in vitro. METTL14 and forkhead box protein 1 (FOXP1) were silenced, and miR-34a-5p was overexpressed in HUVECs to evaluate their effects. HUVEC viability, apoptosis, and levels of intercellular adhesion molecule 1, vascular cell adhesion molecule 1, and endothelin-1 were measured. The N6-methyladenosine (m6A) modification of pri-miR-34a-5p was quantified. The interactions between miR-34a-5p, DiGeorge syndrome critical region 8, and m6A enrichment in miR-34a-5p were analyzed. The relationship between miR-34a-5p and FOXP1 was also verified. The results showed the expressions of METTL14, FOXP1, and miR-34a-5p. METTL14 expression was elevated in the TNF-**α**-induced HUVEC injury model. Silencing METTL14 improved HUVEC viability, inhibited apoptosis, and reduced endothelial inflammation. METTL14 promoted miR-34a-5p expression through m6A modification. Overexpression of miR-34a-5p or silencing FOXP1 reversed the protective effects of METTL14 silencing on cell injury in the PE model. In conclusion, METTL14 mediated m6A modification to promote miR-34a-5p expression, leading to FOXP1 inhibition, which aggravated endothelial cell damage in the PE cell model.

## Introduction

Preeclampsia (PE) is a pregnancy complication characterized by the development of gestational hypertension, proteinuria, and potential liver, kidney, and brain disorders [1]. The global prevalence of PE ranges from 1% to 4%, with wide regional variations [2]. Patients with PE experience placental ischemia and oxidative stress, leading to the secretion of anti-angiogenic factors and pro-inflammatory cytokines by the damaged placenta. This results in endothelial dysfunction and vascular inflammation, eventually causing hypertension and multi-organ damage [3]. Endothelial dysfunction is a key pathological feature at various stages of PE, affecting trophoblast invasion, spiral artery remodeling, and endothelial cell apoptosis [4, 5]. Maintaining a healthy lifestyle during pregnancy is considered a method to reduce the risk of PE, while drug treatments, such as aspirin, antioxidant vitamins, and antihypertensive drugs (e.g., magnesium sulfate) may offer potential therapy, though their effectiveness requires further validation [6]. Thus, the molecular mechanisms of PE need to be further explored to identify more therapeutic options.

N6-methyladenosine (m6A), a chemical derivative of adenosine in RNA, plays a significant role in gene expression, including pre-mRNA processing, mRNA nuclear export, and mRNA stability [7]. m6A modification functions mainly through m6A methyltransferase complexes (“writers”), demethylases (“erasers”), and m6A protein readers. Methyltransferase-like protein 14 (METTL14) is one component of the m6A methyltransferase complex [8]. Studies have shown that METTL14 is highly expressed in diabetic nephropathy, a risk factor for PE, and inhibits α-klotho expression through m6A modification, exacerbating renal pathology, promoting the release of inflammatory factors, and leading to glomerular endothelial cell injury [9]. Importantly, METTL14 has been detected at elevated levels in PE, where it increases circPAPPA2 m6A methylation through m6A modification [10]. However, the downstream effects of METTL14 in PE via m6A modification remain unclear.

MicroRNAs (miRs) are small non-coding RNAs that regulate the transcription of protein-coding genes and participate in various physiological and cellular functions [11]. Upregulation of miR-34a-5p is associated with the release of key inflammatory factors in the pathological process of knee osteoarthritis [12]. miR-34a-5p is significantly enhanced during vascular endothelial cell injury, and its inhibition reduces cell apoptosis and the production of pro-inflammatory factors, thereby playing a protective role in angiogenesis [13]. More importantly, inhibition of miR-34a-5p enhances placental trophoblast cell proliferation, migration, and invasion in PE [14]. However, previous studies primarily focused on miR-34a-5p’s role in regulating trophoblast cell dysfunction [15], with less emphasis on its role in vascular endothelial cell injury in PE.

Forkhead box protein 1 (FOXP1) belongs to the forkhead box protein family and is ubiquitously expressed in normal tissues [16]. A recent study demonstrated that FOXP1 is regulated by upstream miR-218 during the development of PE [17]. FOXP1 expression is inhibited by miR-183, and upregulation of FOXP1 can promote trophoblast proliferation, invasion, and angiogenesis in PE [18].

Maternal vascular endothelial cell injury is a key aspect of PE pathogenesis and is regulated by multiple mechanisms, with inflammation being one of the primary contributors to vascular endothelial cell injury [19]. Tumor necrosis factor alpha (TNF-α) can induce inflammation in the cellular microenvironment to simulate endothelial cell injury [20]. Therefore, based on previous studies [21], we treated human umbilical vein endothelial cells (HUVECs) with TNF-α to mimic PE in vitro and investigate the regulatory role and molecular mechanism of METTL14 in PE, providing new potential treatment strategies.

## Materials and methods

### Cell culture and treatment

HUVECs were purchased from ATCC (Manassas, VA, USA) and cultured in RPMI1640 medium containing 10% fetal bovine serum (with 100 U/mL penicillin and 100 U/mL streptomycin) in an incubator at 37 ^∘^C with 5% CO_2_ and saturated humidity. When cell confluence reached 80%, TNF-α was added at final concentrations of 10, 20, 40, and 80 ng/mL [22] to induce an in vitro vascular endothelial cell injury model, simulating HUVEC injury in PE. The group without TNF-α treatment was used as the control group.

### Cell transfection

Twenty-four hours before TNF-α treatment, si-METTL14-1, si-METTL14-2, si-METTL14-3, si-FOXP1-1, si-FOXP1-2, si-FOXP1-3, miR-34a-5p-mimic, and their corresponding negative controls (Santa Cruz Biotechnology, Santa Cruz, CA, USA) were transfected into HUVECs using Lipofectamine 3000 (Invitrogen, Carlsbad, CA, USA).

### Cell counting kit-8 (CCK-8)

HUVECs were seeded at 5 × 10^3^ cells/well in a 96-well plate, and 10 µL of CCK-8 (Invitrogen) solution was added to the wells after 24 and 48 h of culture, respectively. After 2.5 h of incubation, a termination solution was added, and the absorbance (A) of each well was read at 450 nm using a microplate reader. The cell survival rate of each group was calculated as follows: cell survival rate (%) ═ (A value of the treatment group - A value of the blank group)/(A value of the control group - A value of the blank group) × 100%. The experiment was independently repeated three times.

### Terminal deoxynucleotidyl transferase dUTP nick-end labeling (TUNEL)

The culture medium was removed from treated HUVECs, and cells were fixed in 4% paraformaldehyde for 30 min to prepare cell smears. The cells were incubated with Triton X-100 diluted in 0.3% phosphate-buffered saline for 5 min at room temperature, followed by incubation with TUNEL assay solution (Absin, Shanghai, China) at 37 ^∘^C in the dark for 60 min. The nuclei were counterstained with 4’,6-diamidino-2-phenylindole and observed using fluorescence microscopy. The experiments were independently repeated three times.

### Enzyme-linked immunosorbent assay (ELISA)

The HUVEC supernatants were collected from each group for ELISA detection of intercellular adhesion molecule 1 (ICAM-1), vascular cell adhesion molecule 1 (VCAM1), and endothelin-1 (ET-1). Detection was performed according to the ELISA kit instructions for VCAM1 (ab223591; Abcam, Cambridge, MA, USA), ET-1 (ab133030; Abcam), and ICAM-1 (ab174445; Abcam).

### Quantitative reverse transcription polymerase chain reaction (qRT-PCR)

Total RNA was extracted from HUVECs using TRIzol reagent and reverse-transcribed into cDNA using the Mir-X™ miRNA First-Strand Synthesis Kit or PrimeScript™ RT Master Mix (Takara, Tokyo, Japan). qRT-PCR analysis was performed using SYBR Premix EX Taq (Takara) and a Bio-Rad iQ5 real-time PCR instrument (Hercules, CA, USA). Glyceraldehyde 3-phosphate dehydrogenase (GAPDH) and U6 [23] were used as internal controls for mRNA and miRNA, respectively. The relative expression levels of METTL14, pri-miR-34a-5p, miR-34a-5p, and FOXP1 were calculated using the 2^-ΔΔCt^ method [24]. The primer sequences are shown in Table 1.

**Table 1:** qPCR primers

	**Forward primer (5′-3′)**	**Reverse primer (5′-3′)**
METTL14	GGTTCTGGGGAGGGGTTG	ATGAGGCAGTGTTCCTTTGTTC
pri-miR-34a-5p	TCGGCAGGGGCCAGCTGTGAG	GGCATCTCTCGCTTCATCTT
miR-34a-5p	GCCGAGTGGCAGTGTCTTAG	CTCAACTGGTGTCGTGGA
FOXP1	CGGTACTCAGACAAATACAA	GTCGGAAGTAAGCAAACA
U6	GTGCTCGCTTCGGCAGCA	AAAATATGGAACGCTTCA
GAPDH	AATCCCATCACCATCTTC	AGGCTGTTGTCATACTTC

METTL14: Methyltransferase-like protein 14; FOXP1: Forkhead box protein 1; GAPDH: Glyceraldehyde 3-phosphate dehydrogenase.

### Western blot (WB) assay

HUVECs were lysed on ice with radioimmunoprecipitation assay (RIPA) buffer (Beyotime, Shanghai, China), and protein concentrations were determined using the bicinchoninic acid kit (Beyotime). Protein samples were separated by electrophoresis on 10% sodium dodecyl sulfate polyacrylamide gel electrophoresis (SDS-PAGE) and transferred to polyvinylidene fluoride membranes (Beyotime). The membranes were blocked with 5% skimmed milk for 2 h at room temperature and then incubated with primary antibodies against METTL14 (1:1000; ab252562; abcam), FOXP1 (1:2000; ab93807; abcam), and GAPDH (1:2000; ab9485; abcam) overnight at 4 ^∘^C. Membranes were then probed with a secondary antibody conjugated with horseradish peroxidase (1:2000; ab205718; abcam) for 1 h at room temperature. Protein bands were visualized using an enhanced chemiluminescence kit (Thermo Fisher Scientific, Waltham, MA, USA) and quantified using ImageJ software.

### Quantification of m6A RNA methylation

The m6A content in high-quality RNA was determined using the EpiQuik™ m6A RNA methylation quantification kit (p-9005, Epigentek, Farmingdale, NY, USA). Briefly, total RNA was isolated from cells using TRIzol reagent, and RNA quality was assessed by NanoDrop and 1% agarose gel electrophoresis. The extracted RNA was processed according to the kit instructions. The m6A level was measured by absorbance at 450 nm, and the relative m6A content was calculated using a standard curve.

### RNA immunoprecipitation (RIP)

RIP assays were performed using the Magna RIP kit (Millipore, Billerica, MA, USA) following the manufacturer’s instructions. HUVECs were lysed on ice with RIPA buffer and incubated with an antibody against DiGeorge syndrome critical region gene 8 (DGCR8) (ab90579; Abcam) overnight at 4 ^∘^C to immunoprecipitate endogenous DGCR8. RNA from the precipitate was extracted for qRT-PCR to detect the binding level of pri-miR-34a-5p to DGCR8. For m6A-modified pri-miR-34a-5p detection, the extracted RNA was sonicated after DNase I treatment. Lysed RNA was incubated with beads conjugated to an m6A antibody (ab264408; Abcam) overnight at 4 ^∘^C using the Magna MeRIP m6A kit (Millipore). TRIzol was then used to extract RNA for qRT-PCR analysis.

### Bioinformatics

Targerscan7.2 (https://www.targetscan.org/vert_80/, [25]), DIANA tools (https://dianalab.e-ce.uth.gr/tools, [26]), and miRDB (http://mirdb.org/, [27]) were used to predict downstream target genes of miR-34a-5p. Targerscan7.2 was used to predict the binding sites of miR-34a-5p on FOXP1.

### Dual-luciferase reporter gene assay

FOXP1 wild-type (FOXP1-WT) and mutant (FOXP1-MUT) luciferase plasmids containing the predicted miR-34a-5p binding sites were constructed. FOXP1-WT and FOXP1-MUT plasmids were co-transfected with miR-34a-5p-mimic or NC-mimic into HUVECs using Lipofectamine 3000. Relative luciferase activity was detected using a dual-luciferase reporter assay system (Promega, Madison, WI, USA) 48 h after transfection.

### Ethical statement

This study did not involve any human or animal experiments, only experiments at the cellular level, so ethical approval was not required.

### Statistical analysis

GraphPad Prism 8.0 software (GraphPad Software Inc., San Diego, CA, USA) was used for data analysis. Measurement data are expressed as mean ± standard deviation (mean ± SD). One-way or two-way analysis of variance (ANOVA) was used for multigroup comparisons, followed by Tukey’s post-hoc test. *P* < 0.05 was considered statistically significant.

## Results

### METTL14 was highly expressed in TNF-**α**-induced HUVEC injury

**Figure 1.**
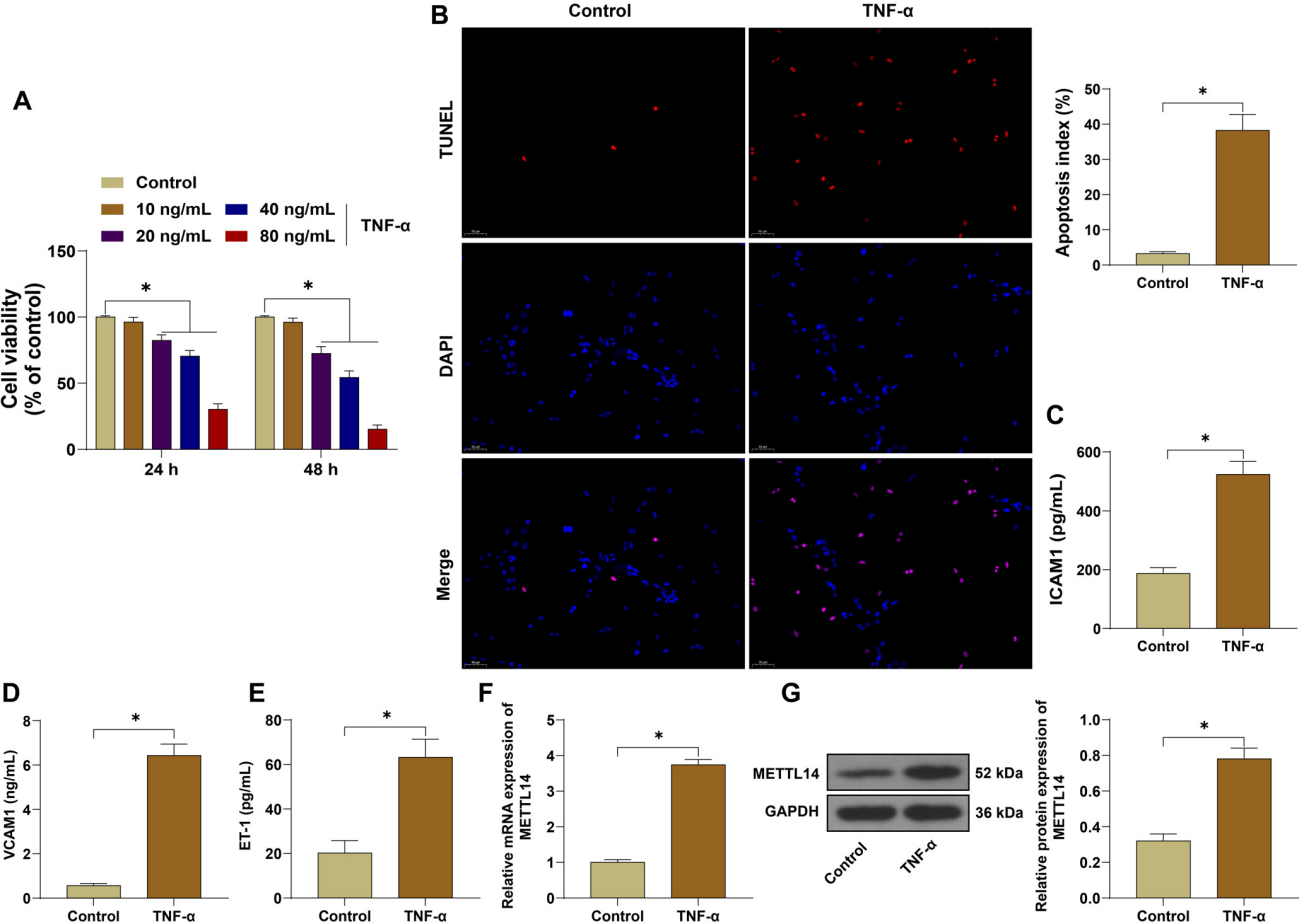
**METTL14 is highly expressed in TNF-α-induced HUVEC injury.** HUVECs were treated with 10, 20, 40, and 80 ng/mL TNF-α. (A) Cell viability at 24 and 48 h post-treatment was determined by the CCK-8 assay; the optimal concentration of TNF-α was 40 ng/mL, and cells were treated for 48 h; (B) Cell apoptosis was assessed by TUNEL assay; (C–E) The levels of ICAM1, VCAM1, and ET-1 were measured by ELISA; (F) METTL14 mRNA expression was detected by qRT-PCR; (G) METTL14 protein expression was determined by WB assay. Independent experiments were repeated three times. Data in panels (B–G) were analyzed by *t*-test, while data in panel (A) were analyzed by two-way ANOVA followed by Tukey’s post-hoc test. *P < 0.05. METTL14: Methyltransferase-like protein 14; TNF-**α**: Tumor necrosis factor-alpha; HUVEC: Human umbilical vein endothelial cells; TUNEL: Terminal deoxynucleotidyl transferase dUTP nick-end labeling; ICAM1: Intercellular adhesion molecule 1; VCAM1: Vascular cell adhesion molecule 1; GAPDH: Glyceraldehyde 3-phosphate dehydrogenase; CCK-8: Cell counting kit-8; ELISA: Enzyme-linked immunosorbent assay; qRT-PCR: Quantitative reverse transcription polymerase chain reaction; WB: Western blot; ANOVA: Analysis of variance; ET-1: Endothelin-1.

To explore the regulatory mechanism of METTL14 in vascular endothelial cell injury in PE, we constructed an in vitro vascular endothelial cell injury model by treating HUVECs with TNF-α. CCK-8 assay results showed that, compared with the untreated control group, 20 and 40 ng/mL TNF-α treatment reduced cell viability in a dose-dependent manner. After treatment with 40 ng/mL TNF-α for 48 h, the inhibition rate of TNF-α on HUVEC viability was close to 50% (Figure 1A, *P* < 0.05). Therefore, cells were treated with 40 ng/mL TNF-α for 48 h in subsequent experiments. TUNEL assay results indicated that the apoptosis rate of HUVECs treated with TNF-α was significantly higher than in the control group (Figure 1B, *P* < 0.05). ELISA results showed that, compared with the control group, the levels of ICAM1, VCAM1, and ET-1 were significantly elevated in TNF-α-treated HUVECs (Figure 1C–1E, *P* < 0.05). qRT-PCR and WB assay results further demonstrated that METTL14 mRNA and protein levels were significantly increased in TNF-α-treated HUVECs (Figure 1F and 1G, *P* < 0.05). These findings suggested that the vascular endothelial cell injury model was successfully constructed in vitro and that METTL14 expression was elevated in injured HUVECs.

### Silencing METTL14 ameliorated TNF-**α**-induced endothelial cell injury

**Figure 2.**
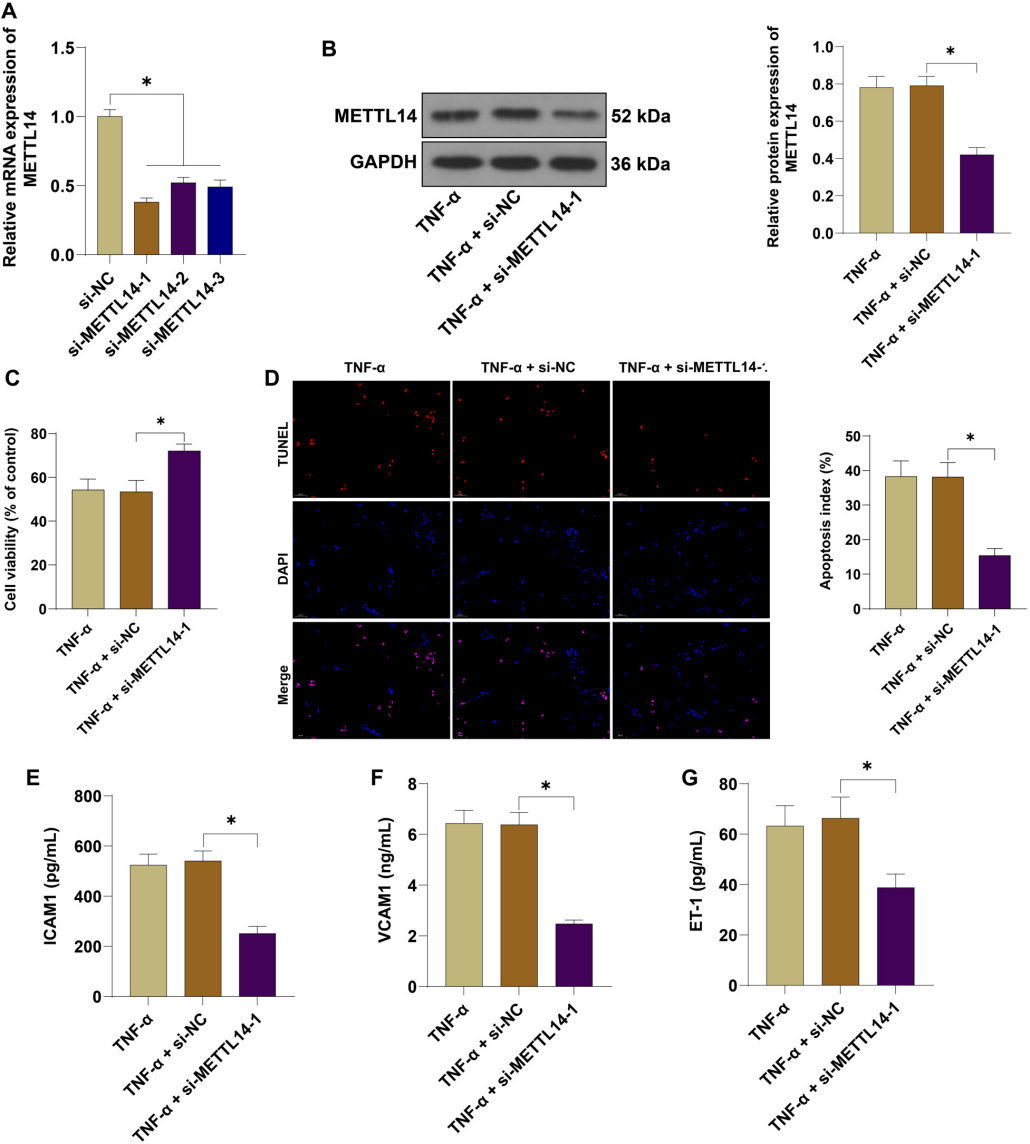
**Silencing METTL14 ameliorated TNF-α-induced endothelial cell injury.** si-METTL14-1, si-METTL14-2, si-METTL14-3, and corresponding negative controls were transfected into HUVECs to silence METTL14 expression. (A) METTL14 mRNA expression was measured by qRT-PCR to verify transfection efficiency; si-METTL14-1-transfected cells were treated with 40 ng/mL TNF-α for 48 h; (B) METTL14 protein expression was measured by WB assay; (C) Cell viability was assessed by the CCK-8 assay; (D) Cell apoptosis was evaluated by TUNEL assay; (E–G) The levels of ICAM1, VCAM1, and ET-1 were measured by ELISA. Independent experiments were repeated three times. Data in panels (A), (B), and (D–G) were analyzed by one-way ANOVA. Data in panel (C) were analyzed by two-way ANOVA followed by Tukey’s post-hoc test. *P < 0.05. METTL14: Methyltransferase-like protein 14; TNF-α: Tumor necrosis factor-alpha; HUVEC: Human umbilical vein endothelial cells; ET-1: Endothelin-1; ICAM1: Intercellular adhesion molecule 1; VCAM1: Vascular cell adhesion molecule 1; GAPDH: Glyceraldehyde 3-phosphate dehydrogenase; qRT-PCR: Quantitative reverse transcription polymerase chain reaction; CCK-8: Cell counting kit-8; WB: Western blot; TUNEL: Terminal deoxynucleotidyl transferase dUTP nick-end labeling; ELISA: Enzyme-linked immunosorbent assay; ANOVA: Analysis of variance.

To investigate the significance of METTL14 in vascular endothelial cell injury in PE, HUVECs were transfected with si-METTL14-1, si-METTL14-2, si-METTL14-3, and corresponding negative controls to silence METTL14 expression (*P* < 0.05, Figure 2A). Cells transfected with si-METTL14-1, which showed the best transfection efficiency, were then treated with 40 ng/mL TNF-α. Silencing METTL14 significantly decreased its protein level (*P* < 0.05, Figure 2B), increased cell viability (*P* < 0.05, Figure 2C), reduced the apoptosis rate (*P* < 0.05, Figure 2D), and lowered the levels of ICAM1, VCAM1, and ET-1 in TNF-α-treated HUVECs (*P* < 0.05, Figure 2E–2G). These results confirmed that silencing METTL14 ameliorated TNF-α-induced HUVEC injury.

### METTL14-mediated m6A modification promoted the expression of mature miR-34a-5p

**Figure 3.**
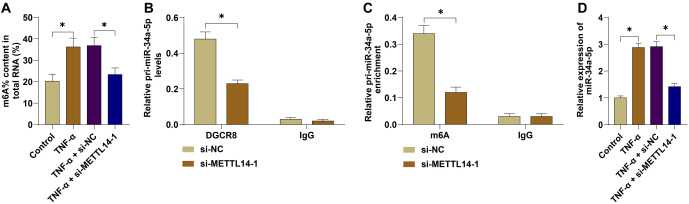
**METTL14-mediated m6A modification promoted the expression of mature miR-34a-5p.** (A) The m6A levels in HUVECs were measured using an RNA m6A quantification assay; (B) The binding level of pri-miR-34a-5p to DGCR8 after immunoprecipitation was determined by qRT-PCR; (C) The level of m6A-modified pri-miR-34a-5p after immunoprecipitation was measured by qRT-PCR; (D) miR-34a-5p expression in cells was detected by qRT-PCR. Independent experiments were repeated three times. Data in panels (A) and (D) were analyzed by one-way ANOVA, while data in panels (B) and (C) were analyzed by two-way ANOVA followed by Tukey’s post-hoc test. *P < 0.05. METTL14: Methyltransferase-like protein 14; HUVEC: Human umbilical vein endothelial cells; TNF-α: Tumor necrosis factor-alpha; qRT-PCR: Quantitative reverse transcription polymerase chain reaction; ANOVA: Analysis of variance.

Next, we explored the mechanism by which METTL14 regulates vascular endothelial cell injury. METTL14 is known to function through m6A modification [28]. We assessed m6A levels in cells and found that the total m6A level was significantly elevated in the TNF-α group (Figure 3A, *P* < 0.05). Silencing METTL14 reduced the total m6A level (*P* < 0.05). Previous studies have reported that METTL14 regulates the m6A modification of miR-34a-5p [29]. We hypothesized that METTL14 may play a role in vascular endothelial cell injury in PE by regulating the m6A modification of miR-34a-5p. RIP assay results showed that silencing METTL14 significantly reduced the level of pri-miR-34a-5p bound to DGCR8 (Figure 3B, *P* < 0.05). MeRIP results further demonstrated that the level of m6A-modified pri-miR-34a-5p in HUVECs was significantly decreased after silencing METTL14 (Figure 3C, *P* < 0.05). qRT-PCR showed that miR-34a-5p expression was significantly increased in the TNF-α group (Figure 3D, *P* < 0.05) but decreased after METTL14 silencing (Figure 3D, *P* < 0.05). These findings confirmed that METTL14 mediates the m6A modification of pri-miR-34a-5p, promoting its binding to DGCR8 and its maturation into miR-34a-5p.

### Overexpression of miR-34a-5p reversed the improvement of METTL14 silencing on TNF-**α**-induced vascular endothelial cell injury

**Figure 4.**
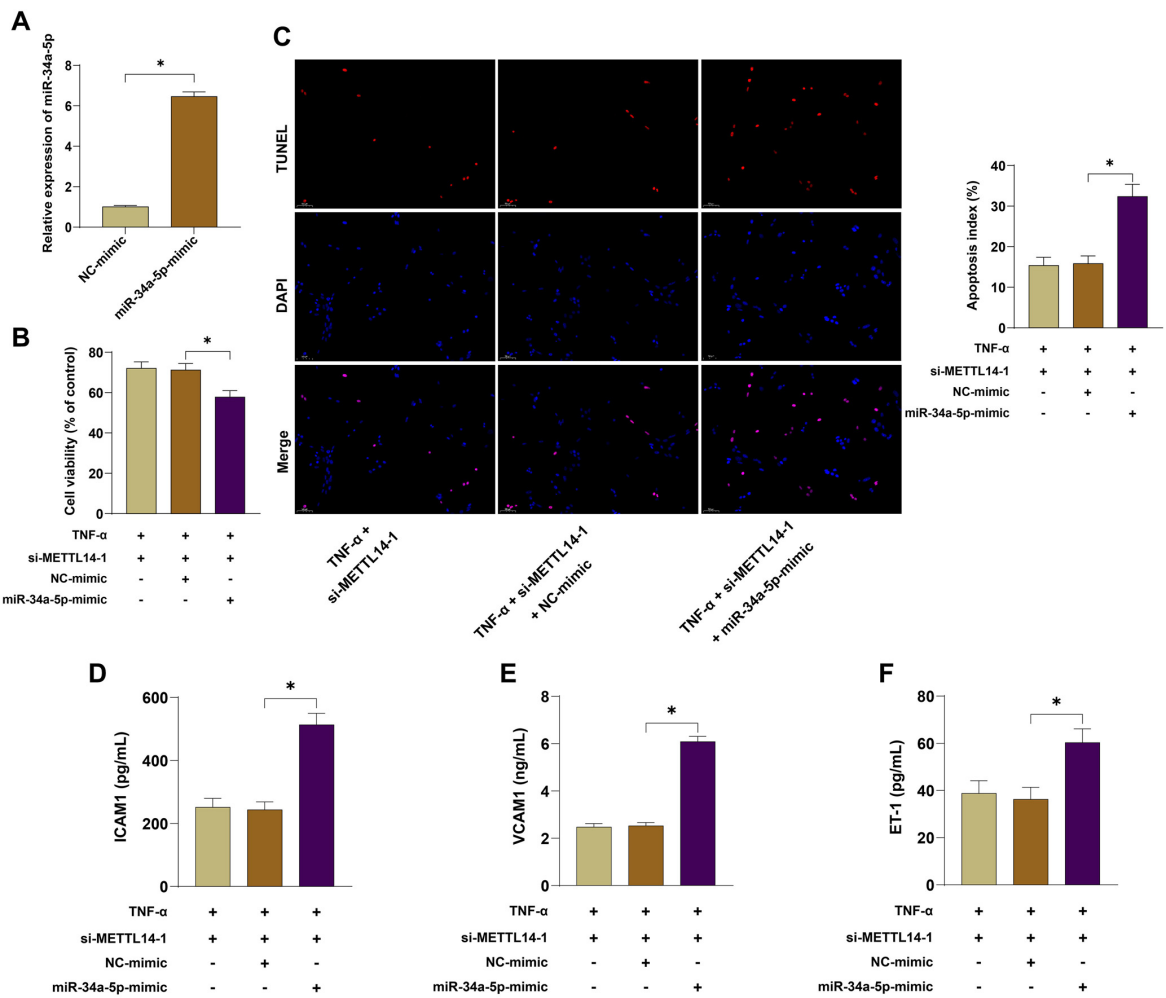
**Overexpression of miR-34a-5p reversed the protective effects of METTL14 silencing on TNF-α-induced vascular endothelial cell injury.** miR-34a-5p mimic and its corresponding negative control were transfected into HUVECs. (A) Transfection efficiency was verified by qRT-PCR; (B) Cell viability was assessed by the CCK-8 assay after treatment with 40 ng/mL TNF-α for 48 h; (C) Cell apoptosis was evaluated by TUNEL assay after treatment with 40 ng/mL TNF-α for 48 h; (D–F) The levels of ICAM1, VCAM1, and ET-1 were measured by ELISA after treatment with 40 ng/mL TNF-α for 48 h. Independent experiments were repeated three times. Data in panel (A) were analyzed by *t*-test, data in panel (B) by two-way ANOVA, and data in panels (C–F) by one-way ANOVA followed by Tukey’s post-hoc test. *P < 0.05. METTL14: Methyltransferase-like protein 14; HUVEC: Human umbilical vein endothelial cells; TNF-α: Tumor necrosis factor-alpha; ET-1: Endothelin-1; ICAM1: Intercellular adhesion molecule 1; VCAM1: Vascular cell adhesion molecule 1; qRT-PCR: Quantitative reverse transcription polymerase chain reaction; CCK-8: Cell counting kit-8; TUNEL: Terminal deoxynucleotidyl transferase dUTP nick-end labeling; ELISA: Enzyme-linked immunosorbent assay; ANOVA: Analysis of variance.

To determine whether METTL14 regulates vascular endothelial cell injury by modulating miR-34a-5p expression, we overexpressed miR-34a-5p and performed combined experiments with si-METTL14-1 (which had the best transfection efficacy), followed by treatment with 40 ng/mL TNF-α (Figure 4A, *P* < 0.05). The results showed that cell viability was significantly reduced in the TNF-α + si-METTL14-1 + miR-34a-5p-mimic group (Figure 4B, *P* < 0.05), while apoptosis was increased (Figure 4C, *P* < 0.05), and the levels of ICAM1, VCAM1, and ET-1 were elevated (Figure 4D–4F, *P* < 0.05). These results indicated that overexpression of miR-34a-5p reversed the protective effects of METTL14 silencing on TNF-α-induced HUVEC injury, suggesting that METTL14 plays a role in vascular endothelial cell injury by regulating miR-34a-5p expression.

### miR-34a-5p targeted and inhibited FOXP1 expression

**Figure 5.**
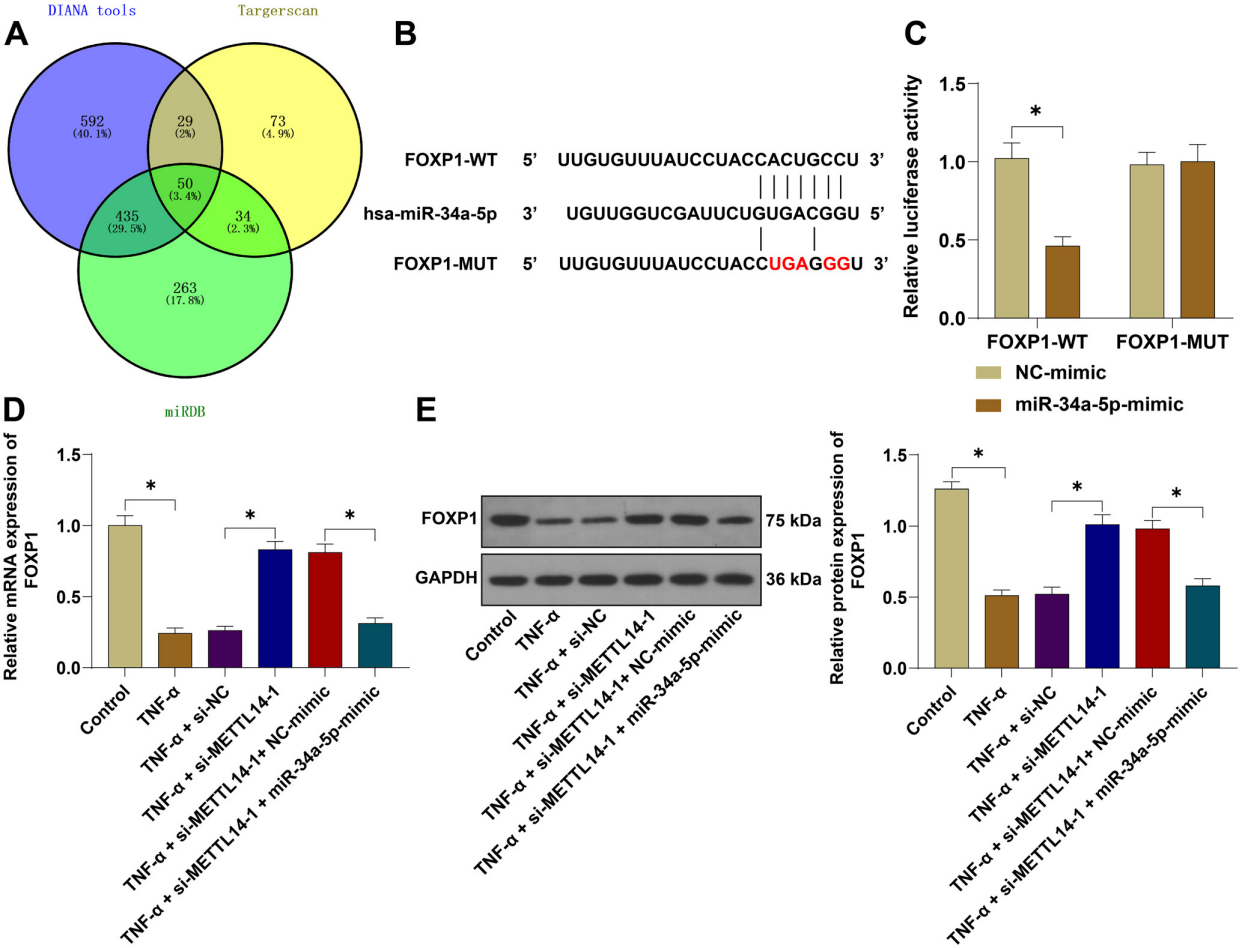
**miR-34a-5p targeted and inhibited FOXP1 expression.** (A) Targetscan7.2, miRDB, and DIANA tools were used to predict the downstream genes of miR-34a-5p; (B) The binding sites between miR-34a-5p and FOXP1 were predicted by Targetscan7.2; (C) The binding relationship between miR-34a-5p and FOXP1 was verified by a dual-luciferase reporter gene assay; (D) FOXP1 mRNA expression in HUVECs was measured by qRT-PCR; (E) FOXP1 protein expression was assessed by WB assay. Independent experiments were repeated three times. Data in panel (C) were analyzed by two-way ANOVA, and data in panels (D and E) by one-way ANOVA followed by Tukey’s post-hoc test. *P < 0.05. FOXP1: Forkhead box protein 1; HUVEC: Human umbilical vein endothelial cells; FOXP1-WT: Forkhead box protein 1 wild-type; FOXP1-MUT: Forkhead box protein 1 mutant; GAPDH: Glyceraldehyde 3-phosphate dehydrogenase; qRT-PCR: Quantitative reverse transcription polymerase chain reaction; WB: Western blot; ANOVA: Analysis of variance.

To explore the downstream mechanism of miR-34a-5p, we predicted its target genes using Targetscan7.2, miRDB, and DIANA tools, and identified FOXP1 as a potential target (Figure 5A). Literature reports have shown that FOXP1 plays a role in PE and vascular endothelial cell injury [18, 30]. The binding sites between miR-34a-5p and FOXP1 were predicted using the Targetscan7.2 website (Figure 5B), and the dual-luciferase reporter gene assay verified the binding relationship between miR-34a-5p and FOXP1 (Figure 5C, *P* < 0.05). qRT-PCR revealed that FOXP1 mRNA expression was significantly reduced in TNF-α-treated HUVECs (Figure 5D, *P* < 0.05), increased after METTL14 silencing, and attenuated after miR-34a-5p overexpression. WB assay results showed a similar trend (Figure 5E, *P* < 0.05). These findings suggest that FOXP1 is a downstream target gene of miR-34a-5p.

### Silencing FOXP1 reversed the improvement of METTL14 silencing on TNF-**α** induced vascular endothelial cell injury

**Figure 6.**
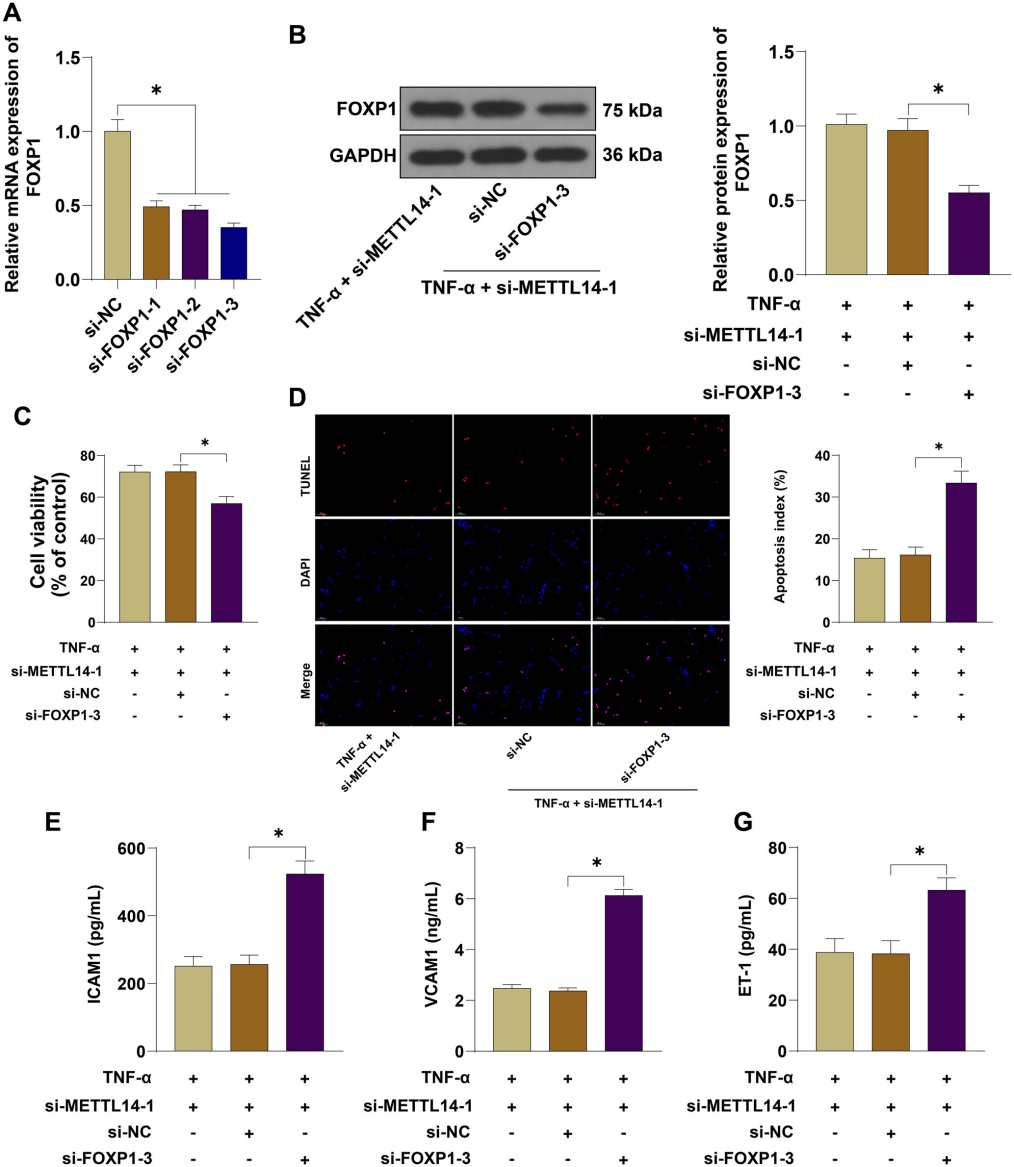
**Silencing FOXP1 reversed the protective effects of METTL14 silencing on TNF-α-induced vascular endothelial cell injury.** HUVECs were transfected with si-FOXP1-1, si-FOXP1-2, si-FOXP1-3, and corresponding negative controls to silence FOXP1. (A) Transfection efficiency was verified by qRT-PCR, and a combined experiment was conducted with si-FOXP1-3 and si-METTL14-1; (B) FOXP1 protein expression after treatment with 40 ng/mL TNF-α for 48 h was detected by WB assay; (C) Cell viability was assessed by the CCK-8 assay; (D) Cell apoptosis was evaluated by TUNEL assay; (E–G) The levels of ICAM1, VCAM1, and ET-1 were measured by ELISA. Independent experiments were repeated three times. Data in panels (A), (B), and (D–G) were analyzed by one-way ANOVA, while data in panel (C) were analyzed by two-way ANOVA followed by Tukey’s post-hoc test. *P < 0.05. METTL14: Methyltransferase-like protein 14; HUVEC: Human umbilical vein endothelial cells; TNF-α: Tumor necrosis factor-alpha; FOXP1: Forkhead box protein 1; qRT-PCR: Quantitative reverse transcription polymerase chain reaction; WB: Western blot; CCK-8: Cell counting kit-8; TUNEL: Terminal deoxynucleotidyl transferase dUTP nick-end labeling; ICAM1: Intercellular adhesion molecule 1; VCAM1: Vascular cell adhesion molecule 1; ET-1: Endothelin-1; ELISA: Enzyme-linked immunosorbent assay; ANOVA: Analysis of variance.

Next, HUVECs were transfected with si-FOXP1-1, si-FOXP1-2, si-FOXP1-3, and corresponding negative controls to silence FOXP1 expression (Figure 6A, *P* < 0.05). Combined experiments with si-FOXP1-3 and si-METTL14-1 (which had the best transfection efficacy) were then conducted. After silencing FOXP1, the expression of FOXP1 protein in the TNF-α + si-METTL14-1 + si-FOXP1-3 group was significantly decreased (Figure 6B, *P* < 0.05), cell viability was reduced (Figure 6C, *P* < 0.05), the apoptosis rate was significantly increased (Figure 6D, *P* < 0.05), and the levels of ICAM1, VCAM1, and ET-1 were elevated (Figure 6E–6G, *P* < 0.05). These results suggest that silencing FOXP1 reversed the protective effects of METTL14 silencing on TNF-α-induced HUVEC injury.

## Discussion

**Figure 7.**
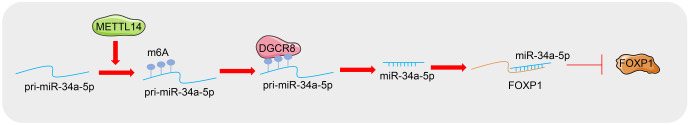
**METTL14-mediated m6A modification promotes the binding of pri-miR-34a-5p to DGCR8, leading to increased expression of mature miR-34a-5p.** miR-34a-5p targets and inhibits FOXP1 transcription and expression, thereby promoting vascular endothelial cell injury in PE. METTL14: Methyltransferase-like protein 14; FOXP1: Forkhead box protein 1; PE: Preeclampsia.

Endothelial injury and systemic inflammatory response are clinical symptoms of PE during pregnancy, often accompanied by inappropriate trophoblast invasion of the myometrium and insufficient spiral artery remodeling, leading to placental ischemia [31, 32]. This suggests that inflammation and endothelial cell injury play crucial roles in the development of PE. This study aimed to investigate the mechanisms of endothelial cell damage in PE, with a focus on METTL14-mediated m6A modification as a potential contributing factor. Our results indicate that METTL14 promotes the processing of pri-miR-34a-5p through m6A modification, increasing the expression of mature miR-34a-5p in the HUVEC injury model. miR-34a-5p was found to target FOXP1, exacerbating HUVEC injury (Figure 7). Silencing METTL14 alleviated HUVEC injury by downregulating miR-34a-5p and upregulating FOXP1 expression.

PE is considered an inflammatory condition associated with elevated expression of endothelial damage markers and inflammatory markers such as TNF-α [33]. Recent studies have confirmed that METTL14 is highly expressed in PE and functions through m6A modification [10, 34]. The mechanism by which METTL14 regulates m6A modification in PE may involve pathways, such as Wnt/β-catenin and mTOR signaling [35]. In this study, we found that METTL14 was highly expressed in TNF-α-induced HUVEC injury, leading to increased apoptosis and elevated levels of intercellular adhesion factors, including ICAM1, VCAM1, and ET-1. Importantly, silencing METTL14 alleviated HUVEC injury. Overexpression of METTL14 has also been shown to aggravate other inflammatory diseases through m6A modification, such as atherosclerosis [36], podocyte inflammatory injury [37], and rheumatoid arthritis [38].

A previous study indicated that METTL14 promotes the maturation of miR-19a by increasing m6A methylation, thereby enhancing the proliferation and invasion of atherosclerotic vascular endothelial cells [28]. Similarly, METTL14 has been shown to regulate the m6A modification of miR-34a-5p [29], which exhibits high expression in PE [15]. Building on this, we considered miR-34a-5p as a downstream regulatory target of METTL14 and explored its role in PE. Previous research has linked miR-34a-5p with three key gene modules in genomic analyses of PE patients [39]. Our findings demonstrate that METTL14 regulates the m6A modification of miR-34a-5p, accelerating its processing from pri-miR-34a-5p to mature miR-34a-5p. Overexpression of miR-34a-5p reduced HUVEC viability, increased the apoptosis rate and pro-inflammatory factors, and aggravated endothelial cell damage. Silencing miR-34a-5p has been shown to enhance trophoblast cell migration and invasion, alleviating PE symptoms [15]. Additionally, overexpression of miR-34a-5p inhibits FOXM1, impairing its protective effects on HUVEC injury and angiogenic activity [13]. miR-34a is abnormally elevated in vascular endothelial dysfunction induced by oxidative stress, while downregulation of miR-34a improves HUVEC survival under oxidative stress [40]. Collectively, this evidence suggests that overexpression of miR-34a-5p exacerbates vascular endothelial cell injury.

FOXP1 has been identified as a downstream target of miRs and plays a role in the progression of PE [17]. Our analysis confirmed the presence of binding sites between miR-34a-5p and FOXP1. As a transcription factor, FOXP1 alleviates endothelial cell dysfunction, apoptosis, inflammation, and oxidative stress by promoting dual specificity phosphatase 12 expression and transcription [30]. FOXP1 also induces trophoblast migration, invasion, and spiral artery remodeling [41–43]. In our study, FOXP1 expression was reduced in TNF-α-treated HUVECs, and silencing FOXP1 significantly aggravated HUVEC injury. FOXP1 has been reported to protect against inflammation and endothelial cell damage, with overexpression reducing vascular inflammation, endothelial inflammasome activation, and endothelial dysfunction [44]. Loss of FOXP1 significantly impairs angiogenesis, endothelial cell proliferation, and migration [45].

There were several limitations to our study. First, we did not investigate the specific role of m6A modification by METTL14 or other regulatory factors in HUVEC injury. Second, we did not conduct animal experiments to assess METTL14’s role in HUVEC injury, which may introduce bias. Third, while TNF-α-stimulated HUVECs are a widely used model for PE [46, 47], this treatment may not fully replicate PE pathology. Therefore, our conclusions based on the cell model require further validation through animal studies and clinical research. Nevertheless, METTL14, miR-34a-5p, and FOXP1 have the potential to serve as therapeutic targets for PE treatment [35, 39, 42, 43]. Based on these findings, we speculate that the METTL14/miR-34a-5p/FOXP1 pathway could serve as a novel therapeutic target for PE. In the future, we plan to: (1) investigate whether METTL14 modifies the m6A modification of other factors involved in endothelial cell injury in PE; (2) include PE patients in studies to assess the expression of METTL14, miR-34a-5p, and FOXP1, and analyze their interactions; (3) establish a PE HUVEC model using serum from PE patients to investigate the regulatory relationship among METTL14, miR-34a-5p, and FOXP1 and their roles in vascular endothelial cell injury; and (4) establish animal models to further verify METTL14’s role in HUVEC injury in PE.

## Conclusion

To sum up, METTL14 expression was increased in the HUVEC injury model. METTL14 promoted the processing of pri-miR-34a-5p through m6A modification, leading to increased expression of mature miR-34a-5p. Silencing METTL14 improved HUVEC viability and alleviated endothelial inflammation by downregulating miR-34a-5p and upregulating FOXP1 expression. In conclusion, regulating the METTL14/miR-34a-5p/FOXP1 pathway has the potential to reduce endothelial cell damage, inflammation, and the risk of adverse maternal and infant outcomes in PE patients. Our findings provide preliminary evidence of the importance of m6A modification in PE, suggesting a potential target for the diagnosis and treatment of PE. These results also offer new insights and directions for further research into the development and progression of PE.

## Data Availability

Data will be made available on request.
